# The Ever-Changing Morphology of Hippocampal Granule Neurons in Physiology and Pathology

**DOI:** 10.3389/fnins.2015.00526

**Published:** 2016-01-19

**Authors:** María Llorens-Martín, Alberto Rábano, Jesús Ávila

**Affiliations:** ^1^Molecular Neurobiology, Function of Microtubular Proteins, Centro de Biología Molecular Severo Ochoa (Consejo Superior de Investigaciones Científicas-Universidad Autónoma de Madrid)Madrid, Spain; ^2^Centro de Investigación Biomédica en Red sobre Enfermedades Neurodegenerativas (Instituto de Salud Carlos III)Madrid, Spain; ^3^Neuropathology Department, CIEN FoundationMadrid, Spain

**Keywords:** neurogenesis, hippocampus, morphology, neurodegeneration, neuroprotection, retrovirus, golgi, newborn granule neuron

## Abstract

Newborn neurons are continuously added to the hippocampal dentate gyrus throughout adulthood. In this review, we analyze the maturational stages that newborn granule neurons go through, with a focus on their unique morphological features during each stage under both physiological and pathological circumstances. In addition, the influence of deleterious (such as schizophrenia, stress, Alzheimer's disease, seizures, stroke, inflammation, dietary deficiencies, or the consumption of drugs of abuse or toxic substances) and neuroprotective (physical exercise and environmental enrichment) stimuli on the maturation of these cells will be examined. Finally, the regulation of this process by proteins involved in neurodegenerative and neurological disorders such as Glycogen synthase kinase 3β, Disrupted in Schizophrenia 1 (DISC-1), Glucocorticoid receptor, pro-inflammatory mediators, Presenilin-1, Amyloid precursor protein, Cyclin-dependent kinase 5 (CDK5), among others, will be evaluated. Given the recently acquired relevance of the dendritic branch as a functional synaptic unit required for memory storage, a full understanding of the morphological alterations observed in newborn neurons may have important consequences for the prevention and treatment of the cognitive and affective alterations that evolve in conjunction with impaired adult hippocampal neurogenesis.

Despite increasing knowledge regarding the developmental steps that control the proliferation, differentiation, and integration of adult-born granule neurons in the hippocampal circuit, the regulatory genes required for morphological maturation and neurite growth of newborn granule cells remain largely unknown. Given that alterations in adult hippocampal neurogenesis (AHN) may be key components in hippocampus-associated neurological diseases, such as major depression (Malberg et al., 2000; Santarelli et al., 2003), schizophrenia (Suh et al., 2013), Alzheimer's disease (AD) (Perry et al., 2012), and epilepsy (Lothman et al., 1992), understanding the molecular mechanisms underlying neuronal migration, neurite extension, and dendrite pathfinding of newborn neurons will be crucial if headway is to be made in the prevention and treatment of neurological and neurodegenerative diseases.

In this review, the fundamental aspects regulating the establishment of the classical morphology of the hippocampal granule neuron will be evaluated under physiological conditions. Pathological aspects will be also discussed. Given the recently acquired relevance of the dendritic branch as a functional synaptic unit needed for memory storage (Govindarajan et al., 2011), alterations in the appropriate branching, structure, and pathfinding of neurites might have far-reaching effects on the synaptic integration and activity of these newborn neurons, phenomena demonstrated to be dysregulated in the aforementioned disorders.

## Variations in the morphology of newborn granule neurons under physiological conditions

### Morphological maturation of newborn granule neurons

**Figure 1:**
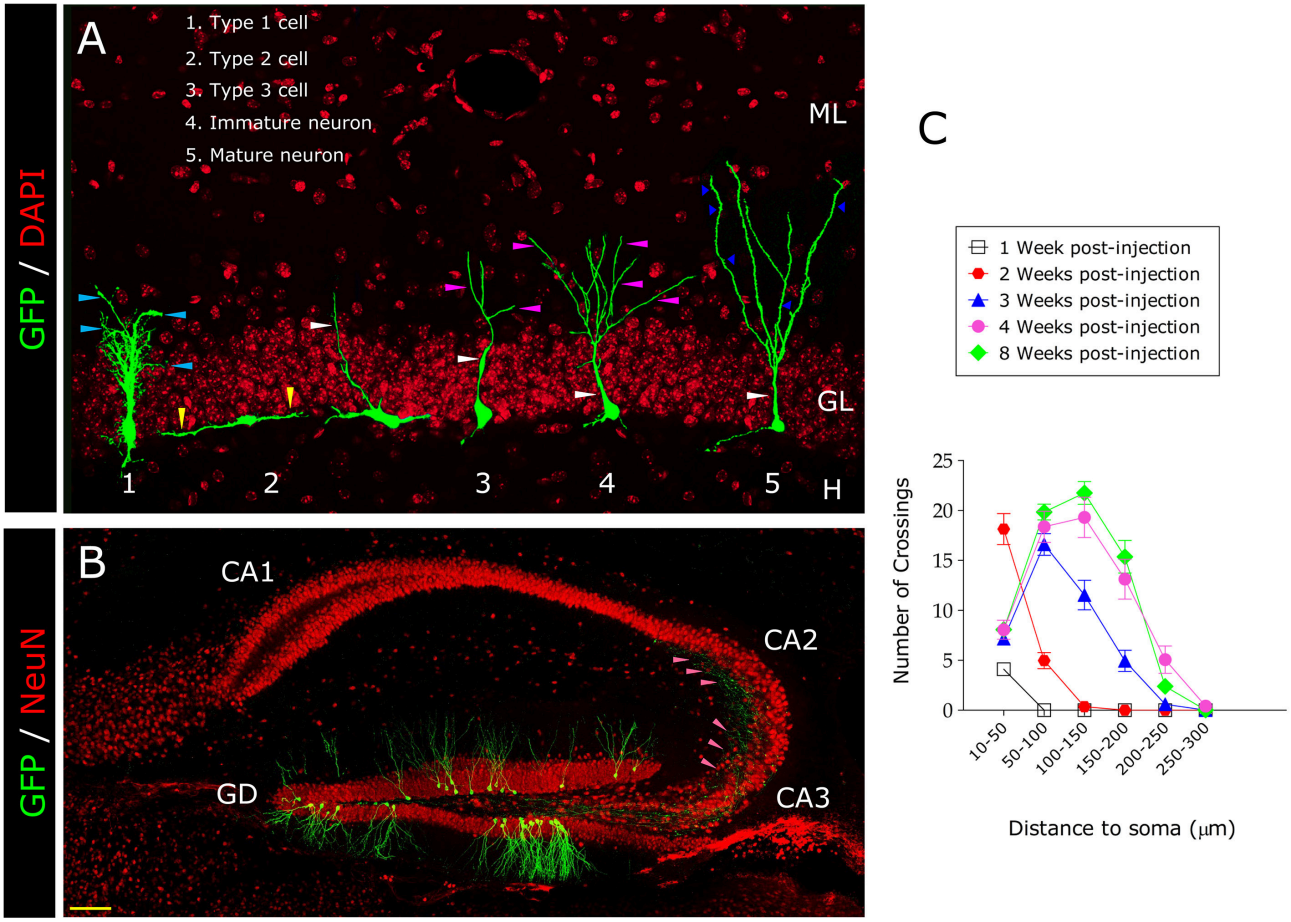
**Morphological maturation of newborn granule neurons under physiological conditions**. In **(A)** the different morphological stages newborn neurons go through before becoming mature are shown. Type-1 cells have a triangular soma and a single apical prolongation that enters the granule cell layer, branching sparsely in the inner molecular layer, where it disperses into many small processes (pale blue triangles). Type-1 cells divide asymmetrically and give rise to transient amplifying progenitors. During their initial stages of differentiation, Type-2 cells have short processes oriented tangentially (yellow triangles) and an irregularly shaped dense nucleus. However, during more advanced stages of differentiation such as the neuroblast stage (Type-3 cells), the greatest morphological changes occur. At the end of this stage, cells are oriented vertically and present a rounded or slightly triangular nucleus and a clearly visible apical dendrite (white triangle). Immature newborn neurons sequentially increase the complexity of their dendritic trees (purple triangles) during subsequent maturational stages until they are indistinguishable from surrounding mature granule neurons. **(B)** Newborn neurons progressively enlarge their axons and send them toward the CA3 and the CA2 regions (pink triangles). In **(C)** the Sholl's analysis of 1-, 2-, 3-, 4-, and 8-, week-old newborn granule neurons is shown. Dendritic branching progressively increases with age. The greatest morphological changes occur between 2 and 4 weeks post-injection. H, Hilus; GL, Granule cell layer; ML, Molecular layer. Yellow scale bar: 100 μm. Dark blue triangles, Dendritic spines.

Under physiological conditions, the generation of newborn neurons in the adult brain of vertebrates occurs mainly in two regions, namely the subventricular zone of the lateral ventricles and the subgranular zone (SGZ) of the hippocampal dentate gyrus (DG) (Kempermann et al., 1998). Adult hippocampal neurogenesis (AHN) occurs in several vertebrate species, including humans (Eriksson et al., 1998; Spalding et al., 2013). In the latter, AHN is quantitatively much more important than adult neurogenesis in the subventricular zone. In addition, while a net age-related reduction of AHN occurs in rodents, in humans only a modest decline in turnover rate has been reported (Spalding et al., 2013), thus supporting the predominant role played by the hippocampus in cognitive processing in our species (Spalding et al., 2013). The maturational stages that newborn neurons go through before becoming fully mature can be identified on the basis not only of the expression of specific molecular markers but also of unique morphological features, as shown in Figure 1A. In the DG, Type-1 cells have a triangular soma and a single apical prolongation that enters the granule cell layer (GL), branching sparsely in the inner molecular layer (IML), where it disperses into many small processes (Kempermann et al., 2004). After dividing asymmetrically, radial-glia-like progenitor (or Type 1) cells give rise to a transiently amplifying population of intermediate neuronal precursors (Type 2 cells). From an electrophysiological and morphological perspective, the transiently amplifying progenitor stage comprises a heterogeneous population of cells. During their initial stages of differentiation (Type-2 cells), they have flabby short processes oriented tangentially and an irregularly shaped dense nucleus. However, during more advanced stages of differentiation, such as the neuroblast stage (Type-3 cells), the greatest morphological and electrophysiological changes occur, and the expression of neuronal markers progressively increases. At the end of this stage, cells are oriented vertically and they present a rounded or slightly triangular nucleus and a clearly visible apical dendrite (Kempermann et al., 2004). By means of retroviral labeling of newborn neurons, Zhao et al. demonstrated that the apical dendrite of these cells reaches the IML and the edge of the molecular layer (ML) at 10 and 21 days post-injection, respectively (Zhao et al., 2006). The complexity of the dendritic tree of immature newborn neurons increases sequentially during subsequent maturational stages. Dendritic spines can be observed for the first time around 16 days after retroviral injection (Zhao et al., 2006). Excitatory synapses appear around the third week of cell life (Kelsch et al., 2008). Once dendritic spines have formed, their number, volume and complexity progressively increase until reaching a plateau at 8–10 weeks of cell age (van Praag et al., 2002). At the end of this maturational process, newborn neurons are fully integrated into tri-synaptic circuits and are electrophysiologically and morphologically indistinguishable from surrounding mature granule neurons (Zhao et al., 2006; Llorens-Martin et al., 2015). Mature granule neurons generally have only one primary apical dendrite emerging from the soma and which is vertically oriented toward the ML. This dendrite remains poorly bifurcated until it reaches the ML, where it branches extensively in order to receive its main afferents, namely the perforant pathway from the Entorhinal cortex (EC). We refer to this characteristic morphology of granule neurons as “Y-shape,” which contrasts with the other shapes present in several pathological conditions, as will be further commented. In this regard, it is noteworthy that, under physiological conditions, the length of the primary apical dendrite is generally inversely correlated to the position that the cell occupies in the GL. Thus, cells whose nuclei are placed in the outer third of the GL have much shorter primary apical dendrites than cells located in the inner section of this layer. While this empirical observation has been systematically reported in the literature, it has received little attention. However, whether migration to the outer sections of the GL is accompanied by and related to the retraction of the primary apical dendrite merits further study. The shortening of the primary apical dendrite should be considered a physiological difference between newborn granule neurons and those generated during development (which are generally located in the outer third of the GL) and should not be confused with the pathological shortening of that occurs in some pathologies. As pointed out by Redila et al., cells with more than one primary apical dendrite appear almost exclusively in the outer third of the GL under physiological conditions and do not seem to correspond to adult-generated neurons but rather to old granule neurons generated during development (Redila and Christie, 2006).

Figure 1 shows the characteristic morphology of the stages of granule cell development previously mentioned (Figure 1A), as well as the representative Sholl's analysis of the dendritic tree of these retrovirally labeled cells at various ages (Figure 1B). As can be observed, the most outstanding outgrowth of the dendritic tree occurs between 2 and 4 weeks after retroviral injections.

During differentiation, newborn neurons progressively lengthen their axons [the mossy fibers (MFs)] and send them toward the CA3 (Zhao et al., 2006) and CA2 (Llorens-Martin et al., 2015) hippocampal regions. Kohara et al. (2014) have recently demonstrated that mature granule neurons establish functional synapses not only with CA3 but also with CA2 pyramidal neurons. We have further confirmed these results and also shown that newborn neurons also establish synapses with the pyramidal neurons in CA2. The time-course of this latter connection follows a similar one to that observed for CA3 (Zhao et al., 2006; Llorens-Martin et al., 2015). The first axonal processes appear in the hilus 10 days after retroviral injection and subsequently reach CA3 and CA2 at 12–13 days (Zhao et al., 2006; Llorens-Martin et al., 2015). Figure 1C shows how the axons of mature granule neurons labeled with GFP-expressing retroviruses reach the CA3 and CA2 regions.

### Differences among mammalian species

As previously commented, the dendritic tree of rodent granule neurons has a “Y-shape.” The lack of basal dendrites in mature granule neurons is a hallmark of these cells in rodents under physiological conditions (Seress and Pokorny, 1981; Shapiro et al., 2005). In fact, granule cells with basal dendrites appear to be a recent formation in phylogeny. In this regard, their morphological variability is greater in humans (Seress and Mrzljak, 1987) than in rats and primates (Seress and Frotscher, 1990; Frotscher et al., 1991; Senitz and Beckmann, 2003). In rats, only up to 2% of granule cells show basal dendrites (Seress and Pokorny, 1981; Spigelman et al., 1998). In primates, 10% of these cells present these structures (Seress, 1992; Seress and Ribak, 1992), while 30% of granule cells from human control subjects show basal dendrites (Seress and Mrzljak, 1987; Senitz and Beckmann, 2003). As an exception, the presence of basal dendrites in rodent granule neurons has been described only in very immature neurons (Ribak et al., 2004) and in organotypic hippocampal cultures. In the latter case, it has been proposed that these structures are due to deafferentation caused by hippocampus sectioning (Heimrich and Frotscher, 1991).

Regarding the morphology of granule neurons in the human brain, in 1987, two simultaneous studies by de Ruiters et al. and Flood et al. were published, in which the morphology of these cells in non-demented aged subjects was described (de Ruiter and Uylings, 1987; Flood et al., 1987). Using Golgi staining, these authors indicated that, as in rodents, inverted-cone morphology is typical of human granule neurons. They showed that the highest branching of the dendritic tree occurs in the inner third of the ML. Although the presence of basal dendrites did not take place in all the subjects studied, the presence of thick basal dendrites full of spines was found to be a common feature of granule neurons. In a later study, Einstein et al. (1994) labeled granule neurons intracellularly with Lucifer Yellow and further confirmed the previous results obtained with Golgi staining, corroborating the presence of numerous spines of distinct shapes in the apical dendrites of these cells. In addition, Senitz et al. described four types of granule neuron in humans on the basis of the positioning and branching of the dendrites (Lauer et al., 2003; Senitz and Beckmann, 2003). In addition, Flood et al. reported age-related dendritic shortening in very elderly humans (Flood et al., 1985, 1987).

**Figure 2:**
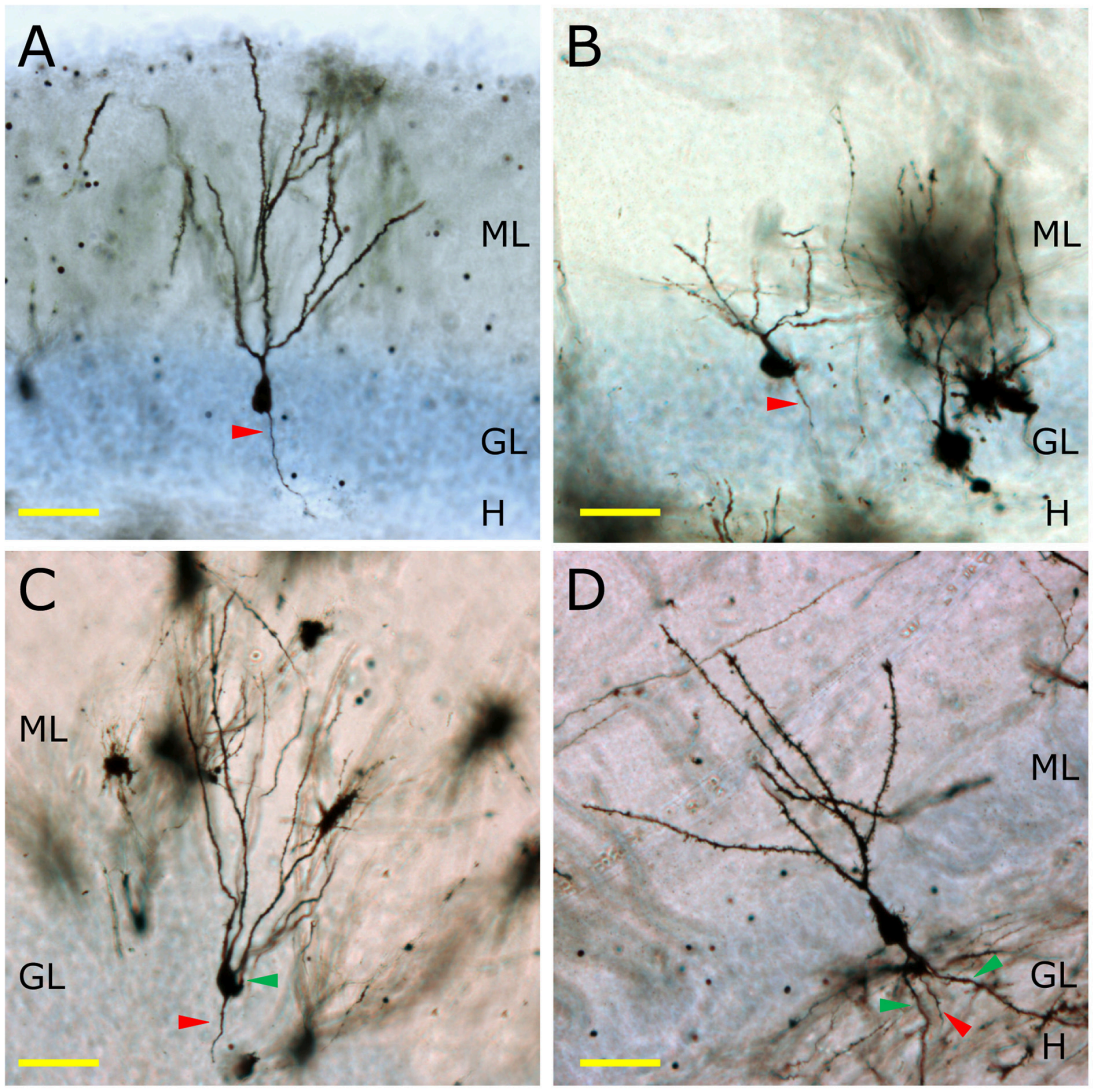
**The morphology of granule neurons among different mammalian species**. In **(A)** the morphology of a Golgi-impregnated murine granule neuron is shown. The total lack of basal dendrites, as well as the presence of one single primary apical dendrite and one axonal process (red triangle) can be observed. In the Capuchine monkey, a great variability of morphologies is observed. A representative neuron lacking basal dendrites and having one apical dendrite and one axonal process is shown **(B)**. Conversely, one of the most remarkable morphological features of the granule neurons of the Chimpanzee **(C)** is the presence of basal dendrites (green triangle). The same feature can be observed in human granule neurons **(D)**, in which a profusely branched basal dendrite emerges from the bottom of the cell soma and ramifies in the hilus. H, Hilus; GL, Granule cell layer; ML, Molecular layer. Yellow scale bar: 50 μm.

In non-human primates, the morphology of granule neurons has received little attention. Kohler et al. reported significant differences in the timing of the maturation of newborn neurons in Macaque monkeys compared to rodents (Kohler et al., 2011). Whereas newborn granule neurons in rodents complete their morphological maturation within 8–10 weeks (Zhao et al., 2006), these authors demonstrated that 4th and 5th branching order dendrites occur only after 11 or 23 weeks of maturation in the newborn granule neurons of the Macaque monkey (Kohler et al., 2011). Figure 2 shows the appearance of the Golgi-stained DGs of the mouse (Figure 2A), Capuchine monkey (Figure 2B), Chimpanzee (Figure 2C), and human (Figure 2D). It should be noted that murine granule neurons lack basal dendrites, whereas chimpanzee and human ones present numerous ramified basal dendrites with abundant spines.

Only a few other species of mammals have received attention regarding the morphology of granule neurons. For example, the granule neurons of megachiropteran bats (flying fox) have been reported to be more similar to those of primates than to those of rodents (Buhl and Dann, 1990). The main difference observed between these bats and rodents is that the latter have functional basal dendrites in granule neurons, while these structures are largely absent in the former under physiological conditions.

## Regulation of the morphological maturation of newborn neurons by neuroprotective stimuli

In general terms, neuroprotective stimuli such as physical exercise or environmental enrichment promote morphological maturation of newborn granule neurons, as will be further discussed in this section.

### Physical exercise

Physical activity is one of the most potent stimulators of AHN (van Praag et al., 1999a,b). It exerts anti-depressant (Bjornebekk et al., 2005; Duman, 2005) and anxiolytic (Duman et al., 2008; Trejo et al., 2008; Salam et al., 2009) actions and stimulates hippocampal-dependent memory (Cotman and Berchtold, 2002; Parle et al., 2005; van Praag et al., 2005; Creer et al., 2010). These beneficial effects have been observed not only in young (van Praag et al., 1999b; Llorens-Martin et al., 2006) but also in aged (Kronenberg et al., 2006; Hollmann et al., 2007; Fabel and Kempermann, 2008) individuals, and physical exercise has been proposed as a co-adjuvant for the treatment of several neurodegenerative and mood disorders (Cotman and Engesser-Cesar, 2002), since it delays the progression of the disease in several animal models (Adlard et al., 2005) and human patients (Cotman and Berchtold, 2002; Hoffmann et al., 2013). Among the hypotheses aiming to explain the beneficial effects exerted by physical activity on the brain, the “neurotrophic hypothesis of physical exercise” postulates that these favorable actions critically depend on the increase in the levels of growth factors that it induces (reviewed in Llorens-Martin et al., 2008). Physical activity increases the levels of brain-derived neurotrophic factor (BDNF) (Neeper et al., 1995, 1996; Oliff et al., 1998), insulin-like growth factor I (Trejo et al., 2001), vascular-endothelial growth factor (VEGF) (Fabel et al., 2003), and nerve growth factor (NGF) (Chae et al., 2012), among others. In the adult hippocampus, running stimulates neuron precursor proliferation (van Praag et al., 1999b; Olson et al., 2006), a process that is mediated by an increase in the circulating levels of IGF-I (Trejo et al., 2001), VEGF (Fabel et al., 2003; During and Cao, 2006), and BDNF (Vaynman et al., 2004). The downstream molecular pathways triggered by these factors converge in the stimulation of the AKT pro-survival pathway and, interestingly, in the inhibition of GSK-3β (Chen and Russo-Neustadt, 2005; Bruel-Jungerman et al., 2009), a pivotal kinase involved in several neurodegenerative disorders (Llorens-Martin et al., 2014b) and that dramatically impairs AHN (Fuster-Matanzo et al., 2013; Llorens-Martin et al., 2013).

Regarding the enhancement of newborn neuron development by physical activity, several studies involving Golgi staining have shown that it increases the percentage of cells with a single primary apical dendrite and also the total dendritic length of granule cells (Redila and Christie, 2006; Stranahan et al., 2007). The use of the same technique revealed that physical activity increases the number of dendritic spines in these cells (Stranahan et al., 2009; Glasper et al., 2010). Using 3R-Tau, a novel marker for newborn neuron axons, we have demonstrated that physical exercise accelerates the appearance of these nerve fibers in newborn neurons (Llorens-Martin et al., 2012) and enhances the innervation of the CA2 region by these cells (Llorens-Martin et al., 2015). Importantly, using a retroviral labeling approach, Zhao et al. demonstrated that physical exercise accelerates the morphological maturation of newborn neurons. In addition to these morphological changes, physical exercise increases the number of mushroom spines in newborn neurons (Zhao et al., 2006).

Of all the neurotrophic factors increased by physical exercise, BDNF seems to play a crucial role in the dendritic alterations caused by running. In fact, BDNF has been demonstrated to exert autocrine actions on the morphological development of newborn neurons, and hence, BDNF knockdown in newborn neurons dramatically reduces the total length and branching of dendrites and prevents the stimulatory effects of exercise on these parameters (Wang et al., 2015). Accordingly, the central knockdown of BDNF causes the same effect on newborn neurons: both the total dendritic length and the number of branches are reduced in mutant mice (Chan et al., 2008). In addition, the overexpression of BDNF leads to an increased dendritic complexity in granule neurons (Tolwani et al., 2002). These observations thus support the pivotal role played by this growth factor in the maturation and integration of newborn granule neurons and emphasize its essential role as mediator of the stimulatory effects of physical exercise.

### Environmental enrichment

Environmental enrichment (EE), an experimental manipulation that consists of a combination of physical activity, social interaction, and cognitive stimulation, is neuroprotective under both physiological and pathological circumstances (van Praag et al., 2000). EE increases AHN both in combination and in the absence of physical activity. In addition to the pro-proliferative actions of physical activity, EE is also a pro-survival stimulus and it triggers the maturation of newborn neurons (Llorens-Martin et al., 2011).

The first studies on the effects of EE on the brain showed that a period of enhanced environmental complexity increases dendritic branching in hippocampal neurons during development (Fiala et al., 1978). In the adult, these studies revealed that EE increased neurite branching and synapse formation in the cortex (Holloway, 1966; Greenough and Volkmar, 1973; Greenough et al., 1973; Diamond et al., 1976). Subsequent research demonstrated that EE triggers similar morphological changes in granule neurons. In particular, this stimulatory procedure increases the number of dendrites per neuron and the complexity of the dendritic tree (Juraska et al., 1985). Using Golgi staining, Faherty et al. showed that EE also increases the total dendritic length of granule neurons (Faherty et al., 2003). By analyzing dendritic complexity in DCX-expressing cells, Choi et al., demonstrated that EE increases the total length and complexity of dendrites in immature neuroblasts (Choi et al., 2008). In addition, these authors further confirmed the notion that BDNF is required for normal dendritic development under standard housing conditions; however, BDNF-independent effects of EE were suggested, since the stimulatory effects of this exposure also occur in BDNF knockout mice (Choi et al., 2008). Using a retroviral labeling approach, we demonstrated that EE increases the maturation and connectivity of newborn granule neurons in control animals and also in a model of AD (Llorens-Martin et al., 2013). These data, together with numerous reports in the literature demonstrating the positive effects of EE on various neurodegenerative and neurological diseases, points to the promise of this approach as a suitable adjuvant treatment for these disorders.

### CREB signaling pathway

One potential mechanism underlying the effects of physical exercise and EE on the dendritic plasticity of newborn granule neurons involves the cAMP response element-binding protein (CREB). Several studies have shown that blocking CREB signaling leads to a dramatic decrease in hippocampal neurogenesis and dendritic arborization in newborn neurons (Jagasia et al., 2009), although the opposite results have also been reported (Gur et al., 2007). Among the many factors activated by CREB cascade, of particular importance are the microRNAs miR-212 and miR-132. The overexpression of miR-132 in cortical neurons increases dendritic branches *in vitro* (Vo et al., 2005). Using a Cre-recombinase-based system, Magill et al. demonstrated that the knockdown of miR-212/132 in newborn granule neurons dramatically decreases dendritic length and branching, and the number of dendritic spines. These authors also reported a drastic distal shrinkage and that most of the neurons lack secondary or tertiary dendrites (Magill et al., 2010).

### Melatonin

Melatonin promotes microtubule polymerization, neuritogenesis, and the formation of dendritic spines *in vitro* (Bellon et al., 2007). In addition, it promotes the survival of newborn neurons in the hippocampus thus contributing to a net increase in the rate of AHN (Ramirez-Rodriguez et al., 2009). A marked decrease in the levels of melatonin has been described both during aging and during the course of neuropsychiatric disorders (Liu et al., 1999; Brusco et al., 2000). Using immunohistochemistry against DCX to label immature newborn neurons, Ramirez-Rodriguez et al. found that chronic melatonin treatment increases the complexity of the dendritic tree of immature neurons. Although melatonin increases dendritic branching in all the domains of the dendritic tree, the changes are more accentuated in the distal parts of the tree, as revealed Sholl's analysis (Ramirez-Rodriguez et al., 2011).

## Regulation of the morphology of newborn granule neurons by detrimental stimuli

Several detrimental stimuli have been demonstrated to be negative regulators of AHN. The particular effects exerted by several of them on the morphology of newborn neurons will be discussed in the following section.

### Epilepsy-induced changes in the morphology of newborn granule neurons

In the nineties, Timm staining was used to show that the MFs of granule neurons of patients with temporal lobe epilepsy (TLE) present a marked sprouting toward the IML (Franck et al., 1995). In addition, von Campe et al. described an increased and aberrant branching of apical dendrites in the IML of TLE patients, as revealed by the injection of Lucifer Yellow into individual granule neurons. It was then proposed that the abnormal morphology of granule neurons contributes to the hyper-excitability of the DG (von Campe et al., 1997). However, whether the morphological features of the individual dysplastic cells give rise to increased susceptibility to seizures or whether these dysplastic cells contribute to seizure activity by establishing abnormal circuits is still a matter of debate. Abnormal basal dendrites, MF sprouting, and recurrent axonal collaterals contacting the dendritic spines of granule neurons have been described in various animal models of epilepsy (Sutula et al., 1988; Holmes et al., 1998, 1999; Dashtipour et al., 2002; Ribak and Dashtipour, 2002; Patel et al., 2004) and in human tissue (Houser, 1992; Lurton et al., 1998; El Bahh et al., 1999). Interestingly, it has been recently described that intrahippocampal kainic acid injection results also in MF sprouting in the CA2 region (Haussler et al., 2015).

These aberrant features are thought to contribute to the establishment of recurrent circuits, which affects the overall balance between excitation and inhibition in the DG (Patel et al., 2004). The abnormal basal dendrites form additional recurrent synapses with the aberrant axonal collaterals of the MF, thus aggravating the recurrent nature of the circuit (Patel et al., 2004). Although not all the experimental data support the idea of individual cell hyper-excitability in epilepsy (Patel et al., 2004), computational (Tejada et al., 2012, 2014) and *in vitro* (Beck et al., 1996; Bausch and McNamara, 2000) models do strongly reinforce this notion. In addition, the electrophysiological properties of granule cells aberrantly located in the hilar region were studied in a rat model of TLE and in epileptic patients by Althaus et al. The authors found an increased excitability in rat neurons whereas neurons obtained from patients displayed a clear reduction in excitability. They affirmed that the discrepancies may reflect differences between the late-stage disease tissue available from human patients and the earlier disease stage examined in the rat TLE model (Althaus et al., 2015). In line with this, Hester et al., found a correlation between severity and duration of seizure and the degree of aberrant integration of newborn granule neurons (namely, their aberrant location, hilar sprouting, and MF loss), thus suggesting that the aberrant synaptic integration of newborn granule neurons directly correlates with epileptogenesis (Hester and Danzer, 2013).

Regarding the specific participation of adult-born neurons to the epileptogenesis of the DG, it has been proposed that only newborn neurons present MF sprouting (Jessberger et al., 2007; Kron et al., 2010) and contribute to the hyper-excitability of the DG. In this regard, it is known that during the development of epilepsy in adult animals, newly generated granule cells integrate abnormally into the hippocampus. These newly generated cells migrate to ectopic locations in the hilus, develop aberrant basal dendrites, contribute to mossy fiber sprouting, and exhibit changes in apical dendrite structure and dendritic spine number (Santos et al., 2011). Using retroviral labeling to visualize newborn neurons, Jessberger et al. described, in a seminal work, a significant number of these cells with aberrant morphology comprising additional basal dendrites directed into the hilus and an ectopic positioning of the cells after seizures (Jessberger et al., 2007). In addition, Cho et al. demonstrated that ablation of neurogenesis is sufficient to alleviate the cognitive decline produced by seizure activity and to prevent the development of subsequent seizures for at least 1 year. This observation thus supports the prevalent role of these cells in the epileptogenesis of the DG (Cho et al., 2015). Moreover, Hester et al., suggested that the accumulation of aberrantly generated newborn neurons directly and specifically contributes to the development of epilepsy (Hester and Danzer, 2013). Interestingly, Santos et al., demonstrated that the apical dendrites of mature newborn neurons generated 2 months before the induction of the status epilepticus, do not undergo morphological changes when they were exposed to *status epilepticus*, although synaptic rearrangement was observed (Santos et al., 2011).

Regarding the morphological changes affecting the apical dendrites of newborn neurons, doublecortin (DCX)-expressing neuroblasts undergo changes in the pilocarpine model of epilepsy (Parent et al., 1997; Arisi and Garcia-Cairasco, 2007). An increased branching of apical dendrites has been shown to occur in the regions in which MF sprouting is most evident, namely the GL and the IML (Arisi and Garcia-Cairasco, 2007). In addition, Overstreet et al. demonstrated that seizures accelerate the morphological maturation and functional integration of 2-week-old newborn neurons into the trisynaptic circuit (Overstreet-Wadiche et al., 2006). Nevertheless, Gao et al. have recently demonstrated that newborn neurons generated 5 days after pilocarpine injection were morphologically indistinguishable from control neurons, although an increase in the percentage of mushroom spines were found in pilocarpine-treated neurons (Gao et al., 2015). The normal integration of newborn neurons generated after status epilepticus into the trisynaptic circuit was further confirmed by the use of retroviral tracing in a work published by Hu et al. (2015).

An finally, in contrast to the data involving newborn neurons in the development of epilepsy, other data suggest that a reduction in neurogenesis increases brain susceptibility to the effects of kainic acid (Iyengar et al., 2015) and that neurogenesis plays a protective role in epileptogenesis (Kempermann, 2006).

Taken together, these data point to the tightly regulated role of newborn neurons in epilepsy. Further studies will be needed to elucidate the individual contribution of cells with varying degrees of maturation to this process. Although it is known that the abnormal networks in which newborn neurons are involved during epilepsy promote abnormal neuronal firing and hyperexcitability, it has yet to be established whether they directly contribute to seizure generation (Hester and Danzer, 2013). In addition, an inspiring idea has been suggested by Murphy et al. (2011). The authors proposed the existence of heterogeneous morphological and synaptic adaptations among the different newborn granule neurons to epilepsy. They observed that, while some newborn neurons underwent certain types of morphological and functional adaptations, other neurons showed the opposite changes, thus suggesting that newborn granule neurons may play diverse homeostatic and contradictory roles in epileptogenesis (Murphy et al., 2011).

### Schizophrenia

Abnormal neuronal development and function of the DG have been proposed as risk factors for schizophrenia (Kobayashi, 2009). Neuropathological studies of patients with schizophrenia revealed cytoarchitectural disturbances in the DG and CA3 regions, including impaired dendritic arborizations (Christison et al., 1989; Arnold et al., 1995) and alterations in synaptic density and MF terminal structure (Kobayashi, 2009).

DISC-1 (Disrupted in Schizophrenia 1) protein, a molecule that determines susceptibility to schizophrenia, is crucial during embryogenesis due to its stimulatory actions on the non-canonical Wnt signaling pathway and its inhibition of glycogen synthase kinase 3-β (GSK-3β) (De Rienzo et al., 2011; Lipina et al., 2011). GSK-3β inhibition by DISC-1 is pivotal for the regulation of AHN (Mao et al., 2009; Ming and Song, 2009). DISC-1 regulates newborn neuron morphology, the synaptic integration of these cells into the trisynaptic circuit (Duan et al., 2007), and their migration within the GL (Meyer and Morris, 2009; Namba et al., 2011). In contrast to the widespread expression of this protein during development, in the adult brain it is greatly restricted, being particularly high in hippocampal granule neurons and the interneurons of the olfactory bulb (Austin and Buckmaster, 2004). DISC-1 knockdown in newborn neurons by means of a retroviral strategy leads to soma hypertrophy, accelerated aberrant dendritic outgrowth with the appearance of ectopic dendrites, overextended migration, enhanced intrinsic excitability, and accelerated synapse formation (Duan et al., 2007). DISC-1 knockout neurons exhibit multiple primary dendrites and show basal dendrites (Duan et al., 2007). In addition, the use of a mouse with a truncated lesion in endogenous DISC-1 revealed that newborn neurons present deficits in axonal targeting in CA3, altered excitability, and increased levels of cAMP (Kvajo et al., 2011). As further demonstrated, some of these morphological alterations are rescued by genetic inactivation of GSK-3 (Lee et al., 2011).

### Stress-exposed granule neurons show altered morphology

The stress-induced increase in the levels of glucocorticoids (GCs) serves many beneficial homeostatic functions (Frank et al., 2013). However, dysregulation of the GC system is associated with cognitive impairments and depression (Snyder et al., 2011). At the cellular level, GCs regulate numerous central processes such as cell proliferation, survival, and death. Due to the involvement of this structure in stress-related neurological diseases, the hippocampal regulation of these processes by GCs has been studied both during development (Gould et al., 1991; Tanapat et al., 1998) and adulthood (Gould et al., 1992; Cameron and Gould, 1994; Gould and Tanapat, 1999).

Glucocorticoid receptor (GR) is highly expressed by hippocampal neurons, especially by CA1 and CA2 pyramidal and granule neurons (Nishi et al., 2007). In addition, the time-course of GR expression during AHN has been addressed by several groups (Gould et al., 1992; Garcia et al., 2004; Llorens-Martin and Trejo, 2011) and has been demonstrated to occur in immature neurons from 1 week after cell birth and onwards (Llorens-Martin and Trejo, 2011). Although a strong dependence of GC effects on the type, duration and strength of the stressor has been observed, in general terms, the stress-induced increase in GCs is thought to suppress AHN (Gould et al., 1997; Kim et al., 2005; Mitra et al., 2006; Llorens-Martin and Trejo, 2011; Ortega-Martinez and Trejo, 2015). Interestingly, AHN has been made responsible for buffering the effects of stress on various behaviors, and its abolishment impairs the restoration of normal levels of GCs after exposure to stress (Opendak and Gould, 2011; Snyder et al., 2011).

However, adrenalectomy (ADX) has been described to exert equivalent suppressive effects on AHN (Liposits et al., 1997; Trejo et al., 2000), thus suggesting that physiological levels of GCs are required for the proper development of hippocampal neurons. Gould, Woolley and McEwen (Gould et al., 1990; Woolley et al., 1990) were the first to report that ADX induces dendritic remodeling in the hippocampus. Golgi staining revealed a shrinkage of the dendritic tree and a reduction in the number of branches per cell 7 days after surgery (Gould et al., 1990). These results are further supported by other studies (Liposits et al., 1997).

In addition, data from other groups unveiled that prenatal stress causes a decrease in the total dendritic length and branching of granule neurons. These changes were observed during adulthood, thus supporting the permanent character of the effects of GCs on granule neurons. A net reduction in distal branching is the most outstanding feature of these alterations (Hosseini-Sharifabad and Hadinedoushan, 2007). Furthermore, the crucial role played by GCs in regulating the temporal dynamics of AHN was demonstrated by Fitzsimons et al. several years ago. They found that the knockdown of GR in hippocampal precursor cells accelerates the morphological maturation, migration into the GL, and functional integration of newborn neurons in the hippocampal circuit (Fitzsimons et al., 2013), thus confirming the specific functions played by GCs in the morphological and synaptic maturation of newborn neurons. Interestingly, the regulation of AHN exerted by GCs appears to have the same inverted “U-shape” characteristic of most of GC actions on the adult brain (Joels, 2006).

### Inflammation

The inflammatory micro-environment strongly influences the survival, maturation, and recruitment of newborn neurons into behaviorally relevant circuits (Belarbi et al., 2012). In this regard, it has been hypothesized that the negative effects of inflammation on hippocampal-related memory are caused by alterations in AHN (Belarbi and Rosi, 2013; Enciu and Popescu, 2013; Kohman and Rhodes, 2013). Microglia, the population of resident macrophage cells within the brain, play continuous role of surveillance, phagocytosis and chemoattraction, among other functions, under both pathological and physiological conditions (Sierra et al., 2013). Although strongly dependent on the type and duration of the pro-inflammatory stimulus, neuroinflammation is considered to impair AHN (Monje et al., 2003; Biscaro et al., 2012). However, the interaction between microglia and newborn neurons is multi-faceted, as both neuroprotective and suppressive effects have been demonstrated (Sierra et al., 2013; Shigemoto-Mogami et al., 2014). In addition, neuroinflammation is a hallmark shared by numerous psychiatric and neurodegenerative diseases, such as schizophrenia (Khandaker and Dantzer, 2015), epileptic seizures (Johnson et al., 2011), depression (Dantzer et al., 2008), and AD (Meraz-Rios et al., 2013). It is thought that microglia orchestrate the transition between innate and adaptive immune responses in the brain (Town et al., 2005). In this respect, the phenotype expressed by microglial cells under either acute or chronic neuroinflammation differs (Boche et al., 2013). Microglia acquire a rapidly induced so-called “M1 classically activated” phenotype when acutely exposed to a pro-inflammatory stimulus (Varnum and Ikezu, 2012). When biased toward this phenotype, these cells secrete pro-inflammatory and chemoattractant mediators (Llorens-Martin et al., 2014a). In contrast, under chronic low-grade inflammation, microglia acquire a “M2 alternatively activated” phenotype (Varnum and Ikezu, 2012), which is characterized by the secretion of both pro- and anti-inflammatory cytokines. This phenotype is considered to be neuroprotective for newborn neurons. In most neurodegenerative diseases, microglial cells are skewed toward the M2 phenotype (Llorens-Martin et al., 2014a).

Using Golgi staining, Jurgens et al. demonstrated that influenza infection causes morphological alterations in hippocampal granule neurons (Jurgens et al., 2012). Influenza induces the retraction and reduces the number of branches in the outer portions of the dendritic tree, whereas it increases total branching near the cell soma. Although the technique used did not allow the determination of the age of the cells studied, the authors described more accentuated morphological and synaptic changes in granule cells located in the inner portion of the GL.

To the best of our knowledge, we were the first to describe that a pro-inflammatory stimulus alters the morphology of newborn granule neurons (Llorens-Martin et al., 2014a). We used the peripheral administration of LPS via subcutaneous osmotic pumps to induce chronic brain inflammation during a range of periods. Using this system in combination with the stereotaxic injection of a Postsynaptic density protein 95 (PSD95) fused to GFP (PSD95-GFP) expressing retrovirus, we subjected newborn neurons to inflammation at various stages of cell maturation and examined its effects. LPS induced striking and long-lasting morphological alterations, namely a marked reduction in distal branching and an increase in proximal branching of the dendritic tree in all the developmental stages studied (Llorens-Martin et al., 2014a). We named this inflammation-induced morphology “V-shape,” due to the presence of several primary apical dendrites in many of the newborn neurons. In addition to these pronounced morphological changes, LPS impaired the connectivity of these cells, thus reducing both afferent (number and size of postsynaptic densities) and efferent (size of the MF terminals) connectivity. These changes in synaptic integration were also long-lasting and, unlike the morphological alterations, they were not fully reversed by an anti-inflammatory treatment with Ibuprofen. This observation suggests that inflammation causes permanent damage to newborn neurons when applied in critical periods of their development.

In the same year, Chugh et al. (2013) described that the intracerebroventricular injection of LPS to retrovirus-injected mice (1 or 4 weeks after retroviral injections) induces changes in the inhibitory synapses produced in newborn neurons. In this case, the authors did not report morphological alterations caused by LPS injection. However, the second stereotaxic injection itself is likely to produce a pro-inflammatory insult strong enough (even in the vehicle-injected animals) to mask some of the putative effects of LPS. It can be hypothesized that this may be the reason why these authors did not observe changes in morphology, although they did describe changes in the migration of newborn neurons (Belarbi and Rosi, 2013; Llorens-Martin et al., 2014a).

### Drugs of abuse

Although numerous studies have focused on the effects of various drugs of abuse on the rate of AHN (reviewed in Powrozek et al., 2004), only a few have examined the consequences of these substances on the morphology of newborn neurons. Ethanol exposure decreases neural stem cell proliferation in the DG (Svanidze et al., 2001; Miki et al., 2003; Powrozek et al., 2004; Redila et al., 2006). However, the effects of ethanol on granule neuron morphology have only recently been addressed. While ethanol exposure increases dendritic complexity in CA3 pyramidal neurons, it reduces the dendritic branching of granule neurons (Staples et al., 2015). In addition, exposure of adult rats to cocaine decreases progenitor cell proliferation in the DG but does not affect the survival or the morphology of newly generated neurons. No changes in dendritic arborization or in positioning in the GL were observed (Dominguez-Escriba et al., 2006; Hauser and Knapp, 2014).

### Neurodegenerative diseases

#### Alzheimer's disease

The most relevant histopathological hallmarks of AD are the extracellular deposits of Amyloid-β (Aβ) and the intracellular tangles formed mainly by hyperphosphorylated Tau protein. The molecular pathways responsible for these two types of aberrant structures converge in a common step, namely the dysregulation of GSK-3β activity (Hernandez et al., 2010). This kinase is one of the most important molecules triggering the hyperphosphorylation of Tau and the subsequent destabilization of the cytoskeleton, Tau aggregation, and neuronal death. Interestingly, GSK-3β is required for the manifestation of the toxic effects of Aβ (Takashima et al., 1993), thus making it one of the most relevant molecules affecting the progression of AD.

It has been widely described that numerous animal models of AD, including GSK-3β-overexpressing mice under the control of the neuronal promoter CamKII, present alterations in the rate of AHN (Fuster-Matanzo et al., 2013). Although the general consensus in the field is that the generation of fully mature and functional neurons is impaired in AD (Li et al., 2008), both increases (Jin et al., 2004a,b), decreases (Crews et al., 2010; Demars et al., 2010; Faure et al., 2011), no changes (Boekhoorn et al., 2006), and changes dependent on the stage of the disease (Chen et al., 2008) have been described in AD patients and animal models. In addition, alterations varying in function of the stage of cell differentiation have been reported in AD patients (Perry et al., 2012; Gomez-Nicola et al., 2014). The in-depth study of the alterations in the AHN rate is not in the scope in this review, which rather focuses on the morphological alterations in granule neurons.

**Figure 3:**
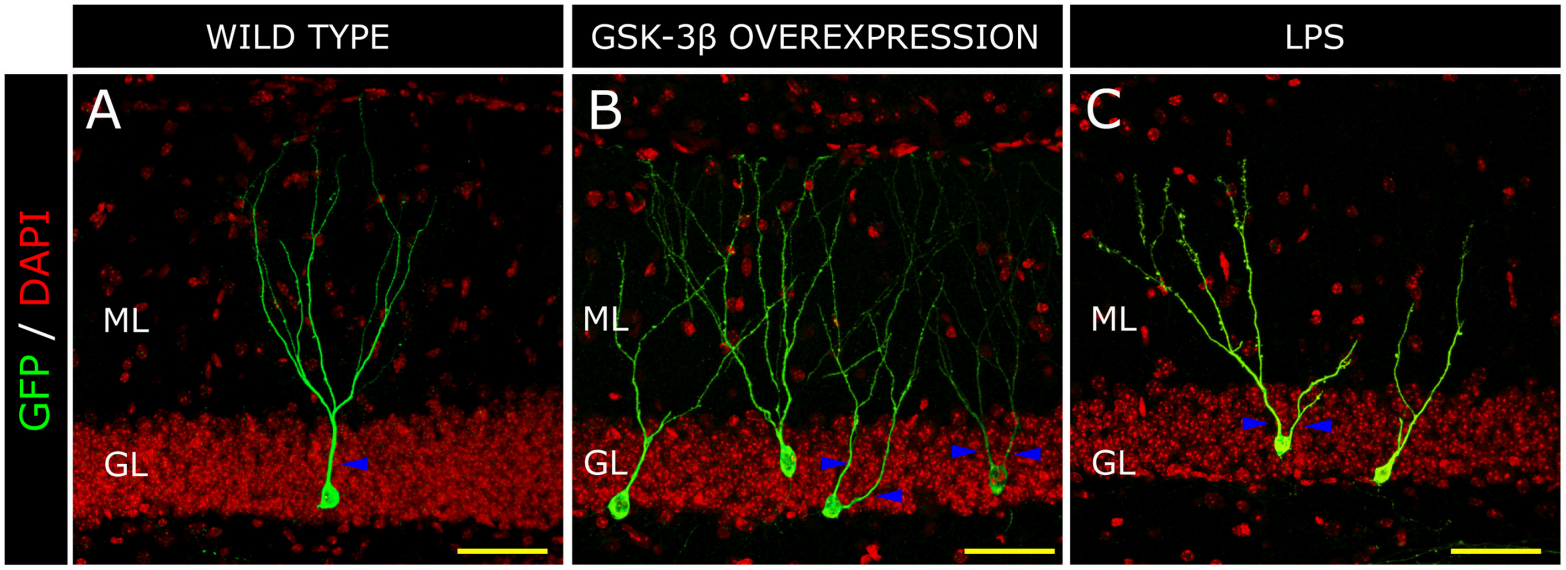
**Morphological alterations in a model of Alzheimer disease (AD) and inflammation**. Retrovirus-labeled newborn neurons from control mice present one apical primary dendrite (blue triangles) and the dendritic tree resembles a “Y-shape” in the control mouse **(A)**. In contrast, in GSK-3β-overexpressing mice (a murine model of AD), cells acquire a “V-shape” morphology, characterized by the presence of several apical dendrites and atrophy of the distal branching of the dendritic tree **(B)**. In a model of brain inflammation induced by the peripheral infusion of LPS, newborn granule neurons also acquire a “V-shape” and show increased branching in the proximal domain of the dendritic tree, whereas distal domains appear to be atrophied **(C)**. H, Hilus; GL, Granule cell layer; ML, Molecular layer. Yellow scale bar: 50 μm.

In 1987, de Ruiter et al., described that granule neurons undergo a profound morphological transformation in advanced stages of AD (de Ruiter and Uylings, 1987). By means of Golgi staining, these authors observed a significant reduction in the length of the distal segments of the dendritic trees of mature neurons. In addition, granule neurons from patients suffering senile dementia were described to present increased branching in the proximal domains of dendritic trees (Flood et al., 1987), in contrast to control newborn neurons which showed a single primary apical dendrite and a “Y-shape” (Figure 3A). It has been proposed that the decreased branching in the outer ML occurs as a result of denervation from the EC or alternatively of aberrant sprouting emerging from cell body (de Ruiter and Uylings, 1987). Given that no differences in the morphological or connectivity alterations were found between neurons located near or distant to the Aβ plaques in AD patients, an Aβ-independent mechanism was put forward to explain the aberrant morphology and reduced connectivity of granule neurons in AD (Einstein et al., 1994). We have proposed that one of these mechanisms might be GSK-3β. The rationale supporting this hypothesis is that the morphological alterations in newborn granule neurons of mice overexpressing GSK-3β are strikingly similar to those described in AD patients (Llorens-Martin et al., 2013; Figure 3B). In this regard, we observed an increased degree of branching in the proximal domain of the dendritic tree and a retraction of the distal domain in the ML. In contrast to the 30% of cells with more than one primary apical dendrite in control subjects, most cells (roughly 90%) in AD patients presented several. Hence, we adopted the terminology of “V-shape” for the granule cells predominant in AD brains. This aberrant morphology might have far-reaching functional consequences, especially taking into account that major afferent connections received by granule neurons arise from the EC and take place within the two outer thirds of the ML (Kharatishvili et al., 2006). Both in these mice and in AD patients, the increased number of proximal branches may favor recurrent connectivity from adjacent granule neurons, whereas the unbranched dendritic tree in the outer ML could account, to some extent, for the DG “disconnection” from the EC, a phenomenon that occurs in AD (Hyman et al., 1984). This disconnection might have important consequences for the function of the hippocampal circuit, since the innervation from the EC drives the dendritic maturation of newborn granule neurons (Frotscher et al., 2000). We have proposed that GSK-3β impairs the maturation of newborn neurons through at least three distinct mechanisms. First, it phosphorylates Tau protein, thereby destabilizing the developing cytoskeleton and its polarity, and hence impairing the establishment of a correct morphology; second, it impairs newborn neuron connectivity by internalizing AMPA receptor GluR1 and triggering the disappearance of synapses (Arendt, 2003; Peineau et al., 2007); and third, it promotes a series of indirect effects linked to neuroinflammation (Jope et al., 2007; Llorens-Martin et al., 2014a), thus aggravating maturational alterations through non-cell-autonomous mechanisms. It has been described, on the one hand, that GSK-3β leads neurons to secrete pro-inflammatory mediators (Martin et al., 2005). On the other, GSK-3β overexpression triggers the death of mature granule neurons (Sirerol-Piquer et al., 2011), a phenomenon that activates microglia and promotes the secretion of more pro-inflammatory cytokines (Pais et al., 2008), thus accentuating the pro-inflammatory nature of the whole process. As previously commented, this pro-inflammatory environment has negative effects on the development of newborn neurons (Belarbi and Rosi, 2013) and is responsible, to some extent, for the maturational alterations found in GSK-3β-overexpressing mice (Llorens-Martin et al., 2014a). In fact, the newborn granule neurons of mice injected peripherally with LPS show a marked “V-shape” (Figure 3C). Thus, whether through direct, cell-autonomous (namely Tau phosphorylation and synapsis removal) or indirect non-cell-autonomous, (neuroinflammation) mechanisms, GSK-3β—a pivotal kinase in AD—dramatically impairs newborn neuron maturation.

However, GSK-3β-overexpressing mice are not the only AD model in which the morphological maturation of newborn granule neurons has been addressed. Using a retroviral labeling technique, Biscaro et al. demonstrated that the expression of familial AD-causing mutations in the amyloid precursor protein and presenilin-1 genes in mice cause a profound impairment of mature granule neuron morphology and connectivity. In this regard, dendritic branching is reduced throughout the dendritic tree and a marked decrease in the number of dendritic spines also occurs in this model (Biscaro et al., 2009, 2012).

Interestingly, CDK5 has attracted great interest due to its involvement in Tau phosphorylation (Maccioni et al., 2001). Similar to the mechanisms proposed to explain the dysregulation of GSK-3β in AD, it has been demonstrated that Aβ also stimulates the activation of CDK5. Thus, inactivating CDK5 prevents the toxic effects of Aβ (Alvarez et al., 1999). GSK-3β and CDK5 are the main kinases phosphorylating Tau and triggering neuronal degeneration in AD (Wen et al., 2008).

CDK5 participates in the signaling pathway of Class 3 Semaphorins (SEM3) (Ng et al., 2013). These molecules play key roles in axonal guidance during development. By silencing the semaphorin receptors neuropilin (NRP) 1 or 2 in newborn neurons, Ng et al., demonstrated a reduction in the length and branching of these cells (Ng et al., 2013). In addition, overexpression of CDK5 in these cells rescues the dendritic phenotypes seen in NRP1- and NRP2-deficient neurons (Ng et al., 2013).

The CDK5 system is formed by the catalytic component (CDK5) and the regulatory proteins P35, P25, and P39 (Maccioni et al., 2001). CDK5 activation in neurons requires association with its regulatory component P35 (Tsai et al., 1994) and this system plays a crucial role both during development and adulthood. CDK5 participates in neuronal migration, neurite extension (Cheung et al., 2007; Ohshima et al., 2007), dendritic pathfinding, synaptic plasticity, and learning (Hawasli et al., 2007; Ohshima et al., 2007). Jessberger et al. used a retrovirus-based approach to overexpress and knockdown CDK5 in newly generated neurons (Jessberger et al., 2008) and to examine the effects of this manipulation on the morphological maturation of these cells. It was found that CDK5 overexpression does not alter the morphology of newborn granule neurons. On the contrary, knocked-down neurons for CDK5 activity lose the typical polarity of granule neurons and extend several dendrites along the GL or even toward the hilus (Jessberger et al., 2008). In addition, they showed a reduced total dendritic length and number of dendritic branches. These morphological alterations appeared from the seventh day after retroviral injections and were maintained thereafter. Interestingly, the knockdown of CDK5 also reduces the number of dendritic spines and the percentage of mushroom spines in these cells (Jessberger et al., 2008). These observations therefore support the prominent role played by this kinase in newborn neuron maturation, as has been further confirmed by other groups (Ng et al., 2013).

#### Synucleopathies: parkinson disease, and lewy body dementia

Synucleinopathies are characterized by a marked cognitive decline, which has a severe impact on the patient's life quality (Possin et al., 2008). Clinical and imaging studies have linked cognitive decline in Parkinson's disease (PD) and Lewy body dementia (LBD) to the malfunction of hippocampal circuitries (Possin et al., 2008; Politis et al., 2010). The most relevant histopathological hallmark of these two conditions is the aggregation of α-synuclein to form Lewy bodies (Spillantini et al., 1997). Impaired AHN has been reported in the brains of PD patients and in murine models of synucleinopathies (Hoglinger et al., 2004). In addition, the expression of α-synuclein in neural progenitors has been observed in PD patients, but not in controls (Winner et al., 2012). Winner et al. demonstrated that the knockdown of α/β synuclein increases the rate of AHN (Winner et al., 2012). In contrast, the overexpression of human α-synuclein in mice dramatically impairs the morphological maturation of newborn granule neurons. In addition, the number of dendritic spines is severely reduced. However, the authors also revealed the cell-autonomous nature of these effects by constructing a retrovirus to overexpress α-synuclein in newborn cells. In this regard, they obtained the same results (Winner et al., 2012).

The morphological alterations in newborn granule neurons of other PD models have also been studied. Mutations in leucine-rich repeat kinase 2 (LRRK2) are considered a risk factor for the development of this disease (Zimprich et al., 2004). Mutation G2019S in Lrrk2 presents the highest genotype- and population-attributable risk (Hulihan et al., 2008). In transgenic mice, the expression of the Lrrk2 G2019S transgene occurs mainly in the neurogenic regions and leads to a drastic reduction in cellular proliferation and survival in the SGZ (Winner et al., 2011). In addition, the morphology of the newly generated neurons is dramatically altered by Lrrk2 G2019S expression. The total length, degree of branching, and branch length of are reduced in G2019S mice (Winner et al., 2011).

### Lead exposure

Epidemiological studies have established a link between ambient air pollutants and health (Verina et al., 2007). Lead is a ubiquitous, highly neurotoxic, environmental and industrial pollutant that particularly affects the developing central nervous system and leads to disease (Block and Calderon-Garciduenas, 2009). This metal has been related to a broad range of physiological, biochemical, and behavioral dysfunctions in humans and in animal models (Ahamed et al., 2009; Dabidi et al., 2013). Chronic exposure to lead impairs synaptic plasticity and cognitive function in animal models (Toscano and Guilarte, 2005; Toscano et al., 2005; Verina et al., 2007). After subjecting adult rats to 2 months of lead exposure, Selvin-Testa et al. reported the appearance of irregular and dark-condensed cytoplasm in hippocampal granule cells. In addition, smooth dendrites, axons, and synaptic terminals were found to be condensed and darker than the surrounding elements (Selvin-Testa, 1991). These authors proposed that these ultrastructural modifications were a morphological substrate for the behavioral alterations caused by exposure to this neurotoxic substance during childhood.

The effects of lead exposure on the maturation of granule newborn neurons have been addressed by Verina et al. They demonstrated that such exposure causes a reduction in both the proliferation and survival of newborn neurons in the hippocampus (Verina et al., 2007). In addition, lead exposure causes a decrease in the length of the dendrites of immature DCX^+^ cells. Specifically, newborn neurons extend dendrites along the GL, and branching in the ML is drastically reduced (Verina et al., 2007). In addition, they also observed a decrease in size of the MF bundle in lead-exposed rats (Verina et al., 2007).

### Dietary factors

It has been demonstrated that a certain prenatal period of malnutrition has adverse effects on spatial memory during adulthood, and the hippocampus has been pinpointed as one of the brain regions most sensitive to nutritional deficits during development. In particular, a reduction in the number of granule neurons and an alteration of the synapse/neuron ratio has been reported in pups of rats subjected to malnutrition during pregnancy (Ahmed et al., 1987). Similar effects have been reported after periods of malnutrition during adulthood (Andrade et al., 1995). In a subsequent study, despite not finding differences in neuron numbers, the same authors reported that food restriction causes a decrease in the total number of dendritic branches of granule neurons. In addition, under these conditions, they observed that the area of the postsynaptic densities in the outer regions of granule neuron dendritic trees tends to decrease (Andrade et al., 2002).

In particular, the effects caused by a deficiency in iodine uptake during development have been systematically studied, since such a deficiency is the most common cause of hypothyroidism and the single most important cause of preventable mental defects (Yu et al., 2014). Iodine deficiency during development downregulates the expression of DCX in the DG (Gong et al., 2010). In iodine-deficient rats, DCX^+^ cells in the DG do not develop dendrites or are significantly shorter than those of control animals (Yu et al., 2014). Accordingly, hypothyroidism during developmental periods leads to a reduction in the number of granule neurons and volume of the DG (Madeira et al., 1991) and to alterations in the morphology of mature granule neurons (Rami et al., 1986), thus supporting the critical role played by the thyroid system in regulating hippocampal development.

On the other hand, it has been proposed that calorie restriction (CR) increases health and lifespan (Levenson and Rich, 2007). Intermittent fasting (IF) has been demonstrated to increase long-term potentiation (LTP) and the expression of the NMDA receptor subunit NR2B in the hippocampus, resulting in enhanced learning (Fontan-Lozano et al., 2007). The molecular mechanisms responsible for the neuroprotective role of CR and IF may be mediated by an increase in AHN in young animals (Lee et al., 2002) and a reduction in the age-related decline in AHN in older animals (Bondolfi et al., 2004). It is thought that both CR and IF increase the survival of neuronal stem cells by boosting the levels of neurotrophic factors (Lee et al., 2002; Bondolfi et al., 2004) and decreasing neuroinflammation (reviewed in Murphy et al., 2014). Despite a prominent increase in the number of dendritic spines in animal models subjected to CR, this regime has not demonstrated to alter the morphology of mature granule neurons (Stranahan et al., 2009), as revealed by means of Golgi staining method. However, specific effects on newborn neurons cannot be ruled out. Furthermore, given the relevant changes induced in both the neurotrophic and inflammatory milieu, it is reasonable to hypothesize that CR has a considerable effect on the morphological maturation of newborn granule neurons. Further studies are required to elucidate this issue.

Interestingly, a high-fat diet decreases the total length and branching of dendrites of newborn neurons, thus drastically impairing the morphological maturation of these cells (Tozuka et al., 2010).

### X-ray irradiation

Exposure of the hippocampus to focal X-ray irradiation during infancy causes a dramatic reduction in granule cell genesis in adulthood (Diaz-Granados et al., 1994; Barlind et al., 2010), thus preventing the MF connection in CA3 (Collet et al., 1999). Furthermore, such exposure impairs hippocampal-dependent learning (Wojtowicz et al., 2008; Hernandez-Rabaza et al., 2009). In addition, it destabilizes the cytoskeleton, leading to morphological alterations in hippocampal neurons (Zhang et al., 2012). Brain inflammation appears to play a key role in radiation-induced abolishment of AHN, since inflammatory blockade restores AHN in a model of cranial irradiation (CI) (Monje et al., 2003). In 2008, Naylor et al. demonstrated that CI alters the angle of orientation of the leading apical process in DCX^+^ cells and that these alterations are reversed by physical exercise (Naylor et al., 2008). In addition, CI during adulthood alters the number and morphology of the dendritic spines in granule neurons. Using Golgi staining, Chakraborti et al. reported a marked reduction in the number and an increase in the proportion of immature, thin and stubby dendritic spines 1 week or 1 month after CI (Chakraborti et al., 2012).

### Stroke

After brain ischemia, the proliferation and differentiation of neuronal progenitors in the DG is strongly stimulated, leading to a significant increase in neurogenesis (Liu et al., 1998; Arvidsson et al., 2001a,b; Kernie and Parent, 2010). Niv et al. used retroviral labeling to visualize newborn neuron morphology and found that stroke leads to a morphologically aberrant integration of adult newborn cells into the DG and that the extent of abnormalities increases with the extent of ischemic damage (Niv et al., 2012). They found that, although most of the neurons had a normally differentiated morphology, a small percentage of newborn granule neurons displayed a marked aberrant morphology, namely bipolar neurons with several basal dendrites, in addition to several long apical dendrites and ectopic neurons located near the hilus (Niv et al., 2012).

## Concluding remarks and further directions

**Figure 4:**
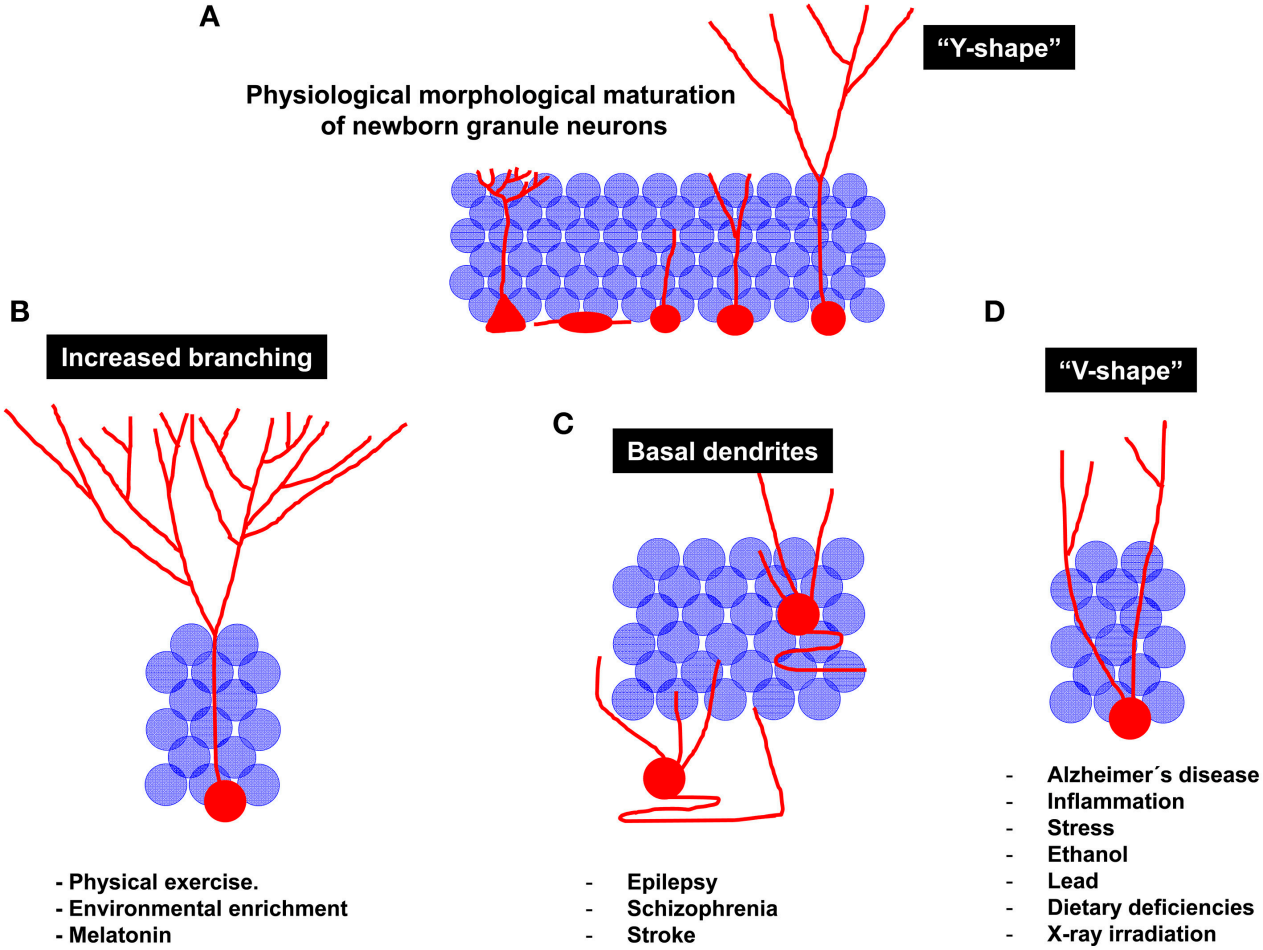
**Schematic diagram of the regulation of newborn granule neuron morphological maturation under physiological and pathological conditions. (A)** Morphological maturation under physiological conditions. It should be noted that, at the end of the maturational process, newborn neurons generally acquire a “Y-shape,” displaying one single primary apical dendrite emerging from the soma. The high dendritic branching observed in the molecular layer lead them to resemble to the aforementioned “Y” shape. **(B)** Neuroprotective stimuli are considered stimulators of newborn neuron maturation. For instance, physical exercise and Environmental enrichment increase dendritic branching of newborn granule neurons. **(C)** Pathological maturation of newborn neurons occurs in epilepsy, schizophrenia and stroke. Under these circumstances, newborn neurons are aberrantly located in the Hilus or in deeper layers of the Granule layer. In addition, recurrent basal dendrites are commonly observed. **(D)** Other pathological circumstances, including neurodegenerative disorders, inflammation, stress, alcohol consumption, dietary deficiencies, lead exposure, or X-ray irradiation result in impaired maturation of newborn neurons. Interestingly, a reduced branching in the molecular layer and the presence of several apical dendrites emerging from the cell soma are observed in these pathological circumstances. These morphological features lead granule neurons to acquire a “V-shape,” in contrast to the “Y-shape” displayed by them under physiological conditions.

In summary, it can be concluded that the physiological process of morphological development comprises drastic modifications in the morphology of the dendritic tree of newborn neurons, namely, a vertical orientation of the apical dendrite and a profuse branching of the apical dendrite in the ML, thus conferring these cells a characteristic “Y-shape” (Figure 4A). The elongation and orientation of the apical dendrite seem to be a process particularly important, on one hand, and particularly vulnerable on the other. Most of the stimuli impairing AHN usually concur in similar morphological alterations of the dendritic tree of granule neurons. For instance, a significant shortening of the primary apical dendrite has been demonstrated to be triggered by inflammation, AD, stress and stroke (Figure 4D), whereas seizures and schizophrenia are known to induce an aberrant positioning and retention og basal dendrites by granule neurons (Figure 4C). In addition, the identity of the primary apical dendrite appears to be lost in most of these pathologies, and cells with more than one primary apical dendrite are commonly found in these conditions, in which granule cells acquire a “V-shape” (Figure 4D). It can be hypothesized that the several apical dendrites present in these “V-shape” neurons after their maturation derive from a defective elimination of the undifferentiated projections present at early maturative stages. Alternatively, a shortening of the primary apical dendrite can result in a “V-shape-like” morphology in these cells. Another crucial unanswered question is whether newborn neurons are equally vulnerable to the different insults during all their maturative stages or whether there are critical checkpoints at which the physiological maturation can be truncated. Intriguingly, an increased migration toward the GL accompanies the shortening of the primary apical dendrite, although these two processes (migration and primary apical dendrite formation) occur at different points of the maturational process. Whether these two processes are causally related or occur only in parallel still remains largely unknown. It can be hypothesized that once the cell migrates toward deeper zones of the GL, the apical dendrite must be shortened to maintain the previously established synaptic contacts. An alternative explanation of these complex phenomena is that the shortening of the apical dendrite is triggered by processes such as cellular aging and oxidative stress. As a consequence of these processes, cytoskeleton destabilization and apical dendrite shortening may trigger the retraction of the nucleus, which would acquire a deeper position in the GL.

Conversely, elements favoring AHN and newborn neuron maturation, such as physical exercise, EE, and melatonin coincide in causing a lengthening of the primary apical dendrite and an increase in branching in the distal domain of the dendritic tree of granule neurons (Figure 4B). Importantly, neuroprotective stimuli, also known to potentiate hippocampal-dependent learning, trigger morphological changes in newborn neurons that drive them toward the perforant pathway, thus giving them greater access to information from the EC. Conversely, insults impairing hippocampal function tend to increase the branching of proximal dendrites aberrantly located in the GL, thereby making newborn neurons more prone to be innervated by and also to contribute to recurrent connections, thus promoting the disconnection of the DC from the physiological hippocampal circuit, a phenomenon known to occur in several neurodegenerative and neurological disorders.

Understanding the molecular mechanisms that underlie the establishment of a correct morphology in newborn neurons is of crucial importance in order to advance in the design of therapeutic approaches to prevent and treat these diseases. In addition, investigating the mechanisms linking morphology and function will be crucial for understanding why this neuronal population undergoing adult regeneration throughout lifetime is so crucial for hippocampal functioning.

## Author contributions

ML did the immunohistochemistry experiments and acquired the optical and confocal microscope images. ML and JÁ wrote the manuscript and obtained necessary funding. AR provided the human samples. ML, JÁ, and AR discussed available data and revised and approved the final version of the manuscript.

## Funding

This study was funded by grants from the Spanish Ministry of Health (SAF-2014-5040-P), and the *Centro de Investigación Biomédica en Red sobre Enfermedades Neurodegenerativas* (CIBERNED, ISCIII; JÁ); and the *Alzheimer's Association* (2015-NIRG-340709; MLL-M).

### Conflict of interest statement

The authors declare that the research was conducted in the absence of any commercial or financial relationships that could be construed as a potential conflict of interest.
